# Some Notes on Phthisis.—II

**Published:** 1908-12-12

**Authors:** 


### SOME NOTES ON PHTHISIS.—II.

### HOARSENESS IN PULMONARY TUBERCULOSIS.

Hoarseness in cases of phthisis pulmonalis is a symptom which may indicate very different conditions. It is apt to be regarded as almost proof of tuberculous laryngitis; unfortunately it is often due to this serious complication of the lung disease, but it is well worth bearing in mind not only that the symptom may occur without there being local tuberculosis, but also that hoarseness may be due to inflammation which is in the pharynx and not in the larynx at all. Dr. Lawrence F. Flick * draws particular attention to this. He points out first of all how difficult it is to obtain satisfactory records of clinical data. At the first blush one would think that to take statistics upon such subjects as hoarseness and the conditions of the tonsils in phthisis cases would be very easy, and that, inasmuch as the throat and the tonsils can be inspected, accurate information about them ought to be attainable. This would all be true if a standard were available for all the conditions upon which statistics have been taken. Unfortunately this is not the case, and what detracts still further from the accuracy of the records is that the statistics have been taken by a large number of men each of whom has his own ideas about what constitutes congestion, inflammation, enlargement, and atrophy. What looks like congestion to one man apparently means a normal condition to another, and what one man terms inflammation another calls congestion. Statistics that are based merely upon a matter of judgment and not upon actual measurement can only be approximately correct.

Much can be learned from Dr. Flick's statistics, however, notwithstanding their necessary imperfections. In the summary for three years, out of 1,882 cases in which the throat condition was recorded, 48 per cent. were set down as having it congested, 13 per cent. as having it inflamed, and 19 per cent. as having it unduly pale. This means that about 80 per cent. of the cases which came for treatment had an abnormal condition of the throat as determined by the eye on careful inspection by trained observers.

Without going into minutiæ it may also be stated that about 20 per cent. of the patients were recorded as having an abnormal condition of the tonsils. Recent investigations show that the pharynx, and especially the tonsils, play a rôle in the implantation of tuberculosis. The data would seem to indicate that they also play an important part in the development and progress of the disease. It is quite possible that the throat and the tonsils by becoming culture-beds for micro-organisms are frequently sources of mixed infection in tuberculosis, and may keep up this mixed infection by supplying new seed to the tissues further down. Secondary inflammations, influenza, and the like are often of the gravest import in a case of latent or arrested phthisis.

The condition of the throat and tonsils should be constantly kept under observation in the treatment of tuberculosis, and every effort should be made to restore them to, and maintain them in, a normal condition. Not only does recovery depend upon this to a very great degree, but protection against recrudescence does so likewise. A patient who has a diseased pharynx and diseased tonsils rarely does well so long as this condition continues, and even when he does make some improvement he is particularly liable to relapse.

Hoarseness in phthisis may be divided into that which is persistent and that which is temporary or intermittent. Persistent hoarseness is usually, though not always, due to some tuberculous lesion in the larynx, whereas temporary hoarseness is more frequently the result of an acute process in the pharynx, or of great debility. Dr. Flick remarks that laryngeal tuberculosis is not necessarily accompanied by hoarseness. The number of cases with persistent hoarseness by no means coincides with the number of cases of laryngeal tuberculosis.

During four years, out of three thousand seven hundred and thirty-three cases of phthisis, 11 per cent. were recorded as having persistent hoarseness, and out of a somewhat smaller total 47 per cent. were recorded as having temporary hoarseness. A comparison of the percentage of cases with congested throats with the percentage of cases with temporary hoarseness shows that the two are pretty nearly equal. There is also a very close relation between the number of cases recorded as having had inflamed throats and those who had persistent hoarseness. This relation is probably more than coincidence. It indicates to some extent that temporary hoarseness is due to a transient condition, while persistent hoarseness is due to injured tissue. Hoarseness, therefore, is not necessarily so grave a symptom in a phthisical patient as might perhaps be supposed.

* *Loc. cit. supra.*

